# Evaluation of rapid diagnostic tests to detect dengue virus infections in Taiwan

**DOI:** 10.1371/journal.pone.0239710

**Published:** 2020-09-29

**Authors:** Li-Teh Liu, Chun-Hong Chen, Ching-Yi Tsai, Ping-Chang Lin, Miao-Chen Hsu, Bo-Yi Huang, Ying-Hui Wang, Jih-Jin Tsai

**Affiliations:** 1 Department of Medical Laboratory Science and Biotechnology, College of Medical Technology, Chung-Hwa University of Medical Technology, Tainan City, Taiwan; 2 National Mosquito-Borne Diseases Control Research Center, National Health Research Institutes, Zhunan, Taiwan; 3 National Institute of Infectious Diseases and Vaccinology, National Health Research Institutes, Zhunan, Taiwan; 4 Tropical Medicine Center, Kaohsiung Medical University Hospital, Kaohsiung, Taiwan; 5 Division of Infectious Diseases, Department of Internal Medicine, Kaohsiung Medical University Hospital, Kaohsiung, Taiwan; 6 School of Medicine, College of Medicine, Kaohsiung Medical University, Kaohsiung, Taiwan; National Yang-Ming University, TAIWAN

## Abstract

Early diagnosis is important for the clinical management of diseases caused by dengue virus (DENV) infections. We investigated the performance of three commercially available DENV nonstructural protein 1 (NS1) rapid diagnostic tests (RDTs) using 173 acute-phase sera collected from dengue fever-suspected patients during the 2012–2013 DENV outbreak in Taiwan. The results of the NS1 RDTs were compared with those of qRT-PCR to calculate the sensitivity and specificity of the NS1 RDTs. The anti-DENV IgM and IgG RDT results were included to increase the probability of detecting acute DENV infection. The anti-DENV IgM/IgG RDT results were also compared with those of IgM/IgG captured ELISA. The sera from DENV qRT-PCR-positive patients were subjected to NS1 RDTs, as well as IgM/IgG captured ELISA. These results suggested that there was no significant difference in the sensitivities of the three commercially available DNEV NS1 RDTs; the SD NS1 RDT results showed the highest agreement with the qRT-PCR reference results, followed in order by the Bio-Rad and CTK NS1 RDT results when the specificity was considered. Inclusion of the IgM or IgG RDT results increased the likelihood of diagnosing either a primary or secondary DENV infection. NS1 RDTs were more sensitive for the detection of primary infections than secondary infections, related to DENV viremia levels determined by qRT-PCR. These results suggested that anti-DENV antibodies reduced the sensitivity of NS1 rapid tests. We also analyzed the sensitivity for the detection of different DENV serotypes, and the results suggested that the NS1 RDTs used in this study were valuable for rapid screening of acute DENV infection with DENV-1, DENV-2 and DENV-3. Our results suggest that the NS1 RDT is a good alternative to qRT-PCR analysis for timely dengue disease management and prevention in dengue-endemic regions where medical resources are lacking or during large dengue outbreaks. However, the relatively low sensitivity for DENV-4 might miss the detection of DENV-4-infected cases.

## Introduction

Dengue is an arthropod-borne acute infectious human disease predominantly distributed in tropical and subtropical countries worldwide, and it is considered the most important arboviral disease in humans [1]. Dengue fever (DF) develops after humans are bitten by female *Aedes albopictus* or *Aedes aegypti* mosquitoes carrying any of the four genetically and antigenically different serotypes of dengue virus (DENV-1 to DENV-4), which were determined by serology studies. DENV is a single-stranded, positive-sense RNA virus that belongs to the genus *Flavivirus*, family Flaviviridae. It is estimated that 390 million DENV infections occur annually, and approximately 2.5 billion people are at risk worldwide. The threat of dengue has grown over the past five decades and continues to increase due to the vast amount of intercontinental travel, travel between urban and rural areas and global warming, resulting in the wide spread of viral vectors [1]. The dengue viral RNA genome (~11 kilobases) encodes seven nonstructural and three structural proteins [2]. The clinical outcomes include asymptomatic virus infection, undifferentiated fever, self-limiting DF, life-threatening dengue hemorrhagic fever (DHF) and dengue shock syndrome (DSS) featuring severe plasma loss characterized by hemostasis abnormalities and increased vascular permeability [3] or dengue, dengue with warning signs, and severe dengue based on the revised WHO case definition [4].

Confirmation of the diagnosis of DENV infection includes DENV isolation, dengue viral genome detection by real-time quantitative reverse transcription-polymerase chain reaction (real-time qRT-PCR) and anti-dengue immunoglobulin M (IgM) detection by IgM-capture enzyme-linked immunosorbent assay (Mac-ELISA) [5]. However, these skills are laborious and take hours to days to perform. Rapid and accurate detection of a DENV infection in acute-phase serum or blood samples from suspected cases could possibly contribute not only to patient management but also to public health by controlling the transmission of the disease. Additionally, the rapid diagnosis of dengue cases at airports and/or harbors in travelers returning from endemic countries could reduce the importation of dengue in countries where dengue it is not endemic, such as Taiwan [6]. NS1 of DENV is associated with dengue genome replication and is detectable from day 1 to day 9 after the fever begins, even when the viral RNA is negative by RT-PCR [7]. The recent development of dengue NS1 antigen detection assays, such as in the format of in-house or commercial ELISA or lateral flow rapid diagnostic tests (RDTs), provides the opportunity for rapid diagnosis of dengue, although the sensitivity and specificity vary between different studies [6–21].

In this study, we evaluated the performance of the three commercial NS1 RDTs using a serum panel collected from 173 donors during the 2012–2013 dengue outbreaks in southern Taiwan using qRT-PCR as a standard method [5].

## Materials and methods

### Study ethics, patients, and sera

The study was approved by the Institutional Review Board (IRB) of the Kaohsiung Medical University Hospital (KMUH) (KMUHIRB-2012-03-14 II and KMUH-IRB-960195). The single acute-phase serum samples used were collected from 173 DF-suspected patients after they signed the informed consent form during the 2012–2013 dengue outbreak in Kaohsiung City, Taiwan. Sera collected during the period between days 0 and 6 post-symptom onset (PSO) were referred to as the acute-phase samples. Serum samples were collected in serum separation tubes (SST, Becton Dickinson, Franklin Lakes, NJ, USA) and stored in aliquots of 200 μl at -80°C. The methods used included the Bio-Rad Dengue NS1 AG Strip, SD Dengue Duo, CTK Dengue Ag and IgG/IgM Combo RDTs, DENV real-time qRT-PCR [22], and an anti-dengue IgM and IgG capture ELISA [23]. All of the experiments were conducted during 2012–2013. A confirmed DENV infection case was defined as a DF-suspected patient associated with a positive qRT-PCR result. The DF-suspected patients with negative qRT-PCR results were defined as having other febrile illnesses (OFIs).

### Viral RNA isolation and one-step SYBR Green-based real-time qRT-PCR

In all of the cases, viral RNAs were extracted from a starting volume of 200 μl of sera using the PureLink Viral RNA Mini Kit (Life Technologies, USA) and immediately subjected to real-time qRT-PCR analysis. Real-time qRT-PCR was performed with a Mx3000P machine (Stratagene, USA). The primer sequences applied for detecting and typing DENV and the formulation of the qRT-PCR have been described elsewhere [22, 24]. Each run included negative controls spiked with water and positive controls spiked with DENV RNA extracted from culture fluid containing DENV. The criteria of a positive control were a threshold cycle (Ct) value of ≤30 and a Tm ≥79°C, while a negative control had a Ct value ≥40 and a Tm <79°C. For the samples, a Ct value of ≤30 or a Tm ≥79°C was considered positive.

### Sequencing

The sequences of amplification products of the conventional serotype-specific RT-PCR were determined using the sequencing service provided by Mission Biotech Company (Taipei City, Taiwan) using a BigDye Terminator v3.1 Cycle Sequencing Kit in a 3730xl DNA Analyzer (Thermo Fisher Scientific Inc., Waltham, MA, USA).

### DENV NS1 protein and anti-dengue IgM/IgG antibody detection using a RDT

Dengue NS1 Ag Strip (Bio-Rad, France), Dengue Ag Rapid Test-Cassette (CTK Biotech, Inc., USA) and SD Dengue Duo (Standard Diagnostics, Inc., Republic of Korea) were used to detect the presence of DENV NS1 antigen in the serum samples (15–25 mins). For rapid detection (15–20 mins) of anti-dengue IgM and IgG antibodies in the sera, Dengue IgG/IgM Combo Rapid Test-cassette (CTK Biotech, Inc., USA) and SD Dengue Duo (Standard Diagnostics, Inc., Republic of Korea) were used. All of the RDTs were performed, and the results were determined following the manufacturers' instructions.

### Anti-dengue IgM and IgG capture ELISA

Anti-DENV IgM and IgG antibodies in the serum samples were detected using the experimental procedures described elsewhere [23] with modifications. The ODs read from the culture fluids of C6/36 cells with a DENV or JEV infection as the antigen were designated as the test absorbance and JEV control values for each sample in the ELISA. For IgM interpretation, a positive sample was defined as having a test absorbance ≥0.5 and a ratio to JEV (as an antigen) ≥2.0, and a negative sample was defined as having a test absorbance <0.4. An OD read from DENV (as an antigen) ≥0.5 and a ratio to JEV (as an antigen) <2 or 0.4≤ OD <0.5 was defined as undetermined. For IgG interpretation, a positive sample was defined as having a test absorbance ≥0.5, and 0.4≤ OD <0.5 was defined as undetermined. In addition, primary DENV infection was defined as the following with a positive qRT-PCR result: i. IgM+/IgG-; ii. IgM-/IgG-; and iii. IgM+ and ratio of the IgM to IgG readings ≥1.20. Secondary DENV infection was defined as the following with a positive qRT-PCR result: i. IgM-/IgG+; and ii. IgG+ and a ratio of the IgM to IgG readings <1.2 [25]. If an IgM or IgG ELISA result was defined as undetermined, the status of primary or secondary infection was defined as undetermined.

### Statistical analysis

The statistical analyses were performed using SPSS software, version 17.0 (SPSS, Chicago, IL, USA). Kappa coefficient values <0 indicated no agreement, and 0–0.20 indicated poor agreement, 0.21–0.40 indicated fair agreement, 0.41–0.60 indicated moderate agreement, 0.61–0.80 indicated substantial agreement, and 0.81–1 indicated almost perfect agreement.

## Results

### Demographic data of enrolled patients

The demographic data of the patients enrolled in the current study (n = 173) are shown in Table 1, and all of the patients were suspected of being DF patients according to the definition proposed by the WHO [4]. The confirmed DF cases were defined by virtue of the positive results of DENV qRT-PCR (n = 136), while those with negative qRT-PCR results were classified as having OFIs (n = 37). The serotype of the DENV was determined by serotype-specific RT-PCR and confirmed by DNA sequencing.

**Table 1:** Baseline characteristics of confirmed dengue and other febrile illness cases in this study.

Variable^a^	Confirmed dengue^b^	Other febrile illness
	N = 136	N = 37
Male sex	74 (55.6%)	18 (48.6%)
Age (years)	47 (12–89)	47 (8–89)
Day of illness	3 (0–7)	3 (0–7)
DENV serotype and serological status^c^		
DENV-1	44 (32.6%)	-
Primary	43 (97.7%)	-
Secondary	0 (0%)	-
Undetermined	1 (2.3%)	-
DENV-2	44 (32.6%)	-
Primary	33 (75.0%)	-
Secondary	5 (11.4%)	-
Undetermined	6 (13.6%)	-
DENV-3	44 (32.6%)	-
Primary	35 (79.5%)	-
Secondary	5 (11.4%)	-
Undetermined	4 (9.1%)	-
DENV-4	3 (2.2%)	-
Primary	3 (100%)	-
Secondary	0 (0%)	-
Undetermined	0 (0%)	-

^a^ Presented as the numbers (%) except for age and day of illness, which are shown as medians (range).

^b^ Confirmed dengue was based on positive DENV qRT-PCR results.

^c^ Sample volume of 1 case was not sufficient for IgM and/or IgG analysis after the samples were subjected to RT-PCR analysis.

### NS1 rapid tests versus reference qRT-PCR analyses

The sensitivities of the three NS1 RDTs tested were not significantly different for acute dengue diagnosis (Table 2). The specificity of the Bio-Rad NS1 rapid test was the highest, while CTK was the lowest among the three rapid tests. Inclusion of the IgM parameter in the CTK and SD RDTs slightly increased the diagnostic sensitivity, although the difference was not significant. Inclusion of the IgM or IgG in the CTK and SD NS1 rapid tests increased the diagnostic sensitivity, but the specificity was decreased, especially for the CTK rapid tests. In addition, combination of the IgM/IgG ELISA results with Bio-Rad NS1 RDT increased the detection sensitivity significantly but not for IgM only. In general, the performance of SD RDT NS1 was the best among the three RDTs tested according to the Kappa value. In addition, combination of the IgM/IgG ELISA results with Bio-Rad NS1 RDT increased the detection sensitivity significantly but not for IgM only.

**Table 2:** Overall performance characteristics of each assay based on the reference qRT-PCR test.

Assay parameter	Total patients (N)	Acute dengue cases^a^ (N)	Number positive (N)	Sensitivity^b^	Specificity^c^	Kappa value^d^	PPV^e^	NPV^f^
SD NS1	173	136	122	89.7% (122/136)	91.9% (34/37)	0.7363 (0.6197–0.8529)	97.6% (122/125)	70.8% (34/48)
Bio-Rad NS1	173	136	116	85.3% (116/136)	94.6% (35/37)	0.6787 (0.5582–0.7992)	98.3% (116/118)	63.6% (35/55)
CTK NS1	173	136	121	89% (121/136)	73% (27/37)	0.5904 (0.4466–0.7342)	92.4% (121/131)	64.3% (27/42)
SD NS1 or IgM RDT	173	136	130	95.6% (130/136)	89.2% (33/37)	0.8314 (0.7305–0.9323)	97% (130/134)	84.6% (33/39)
SD NS1 or IgM or IgG RDT	173	136	132	97.1% (132/136)	86.5% (32/37)	0.8438 (0.7449–0.9427)	96.4% (132/137)	88.9% (32/36)
Bio-Rad NS1 or IgM ELISA^a^	173	136	125	91.9% (125/136)	91.9% (34/37)	0.7769 (0.6663–0.8875)	97.7% (125/128)	75.6% (34/45)
Bio-Rad NS1 or IgM/IgG ELISA^b^	173	136	127	93.4% (127/136)	86.5% (32/37)	0.7685 (0.6534–0.8836)	96.2% (127/132)	78% (32/41)
CTK NS1 or IgM RDT	173	136	130	95.6% (130/136)	70.3% (26/37)	0.6927 (0.557–0.8284)	92.2% (130/141)	81.3% (26/32)
CTK NS1 or IgM or IgG RDT	173	136	133	97.8% (133/136)	43.2% (16/37)	0.4987 (0.332–0.6654)	86.4% (133/154)	84.2% (16/19)

^a^ Based on a positive DENV qRT-PCR result.

^b^ The sensitivity is presented as a % (number of positive NS1 results/number of positive qRT-PCR results).

^c^ The specificity is based on 37 samples from patients with OFIs (negative qRT-PCR results).

^d^ The numbers in parentheses indicate 95% confidence intervals.

^e^ PPV: Positive predictive value.

^f^ NPV: Negative predictive value.

### Sensitivity of NS1 tests by day of illness

We evaluated the difference in the sensitivity of RDTs in different phases of DENV infection using sera samples collected within three days post-symptom onset (PSO) and later (Table 3). The sensitivity of the Bio-Rad NS1 RDTs was lower than that of both the CTK and the SD NS1 RDT with the test specimens collected within three days PSO, although the difference was not significant. Similarly, the sensitivity of the Bio-Rad NS1 RDTs was slightly lower than that with both the CTK and the SD NS1 RDTs in the test specimens collected after 3 days PSO without significance. In general, the sensitivity of all three NS1 RDTs was lower in the test samples collected after three days PSO than in those collected within 3 days PSO. In addition, inclusion of the IgM results alone or with the IgG results resulted in increased sensitivity for both the CTK and the SD RDTs regardless of the time at which the samples were collected.

**Table 3:** Sensitivity of the NS1 RDTs with or without anti-dengue antibodies in plasma samples collected within 3 days of illness onset versus those collected at a later time.

		Bio-Rad NS1	SD NS1	SD NS1 or IgM	SD NS1 or IgM or IgG	CTK NS1	CTK NS1 or IgM	CTK NS1 or IgM or IgG
Status^a^	Total (N)	% Sensitivity						
Collected ≤3 days PSO	69	89.9% (62/69)	95.7% (66/69)	97.1% (67/69)	97.1% (67/69)	95.7% (66/69)	97.1% (67/69)	97.1% (67/69)
Collected >3 days PSO	67	80.6% (54/67)	83.6% (56/67)	94% (63/67)	97% (65/67)	82.1% (55/67)	94% (63/67)	98.5% (66/67)
*P* value^b^		0.151	0.025	0.437	1.000	0.014	0.437	1.000

^a^ PSO: post-symptom onset.

^b^ Fisher's exact test.

### NS1 sensitivity in primary or secondary infection

In primary dengue cases, the sensitivity of the Bio-Rad NS1 rapid tests was 90.4%, and that of both the CTK and the SD NS1 rapid tests was higher than that with the Bio-Rad NS1 tests (Table 4). Inclusion of IgM alone or with IgG parameters in both the CTK and the SD rapid tests did not increase the DENV infection detection sensitivity significantly in primary DENV infections. In secondary dengue cases, the sensitivity of the three NS1 rapid tests was significantly lower than that with the primary dengue cases, but the sensitivity was not significantly different among the three NS1 tests. Inclusion of IgM alone or both IgM/IgG parameters in both the CTK and SD rapid tests increased the detection sensitivity in secondary DENV infections (60% *vs* 90%, P = 0.303; 60% *vs* 100%, P = 0.087). The sensitivities of NS1 rapid tests were influenced by the DENV viremia levels (Ct: 19.42 for primary infection and 29.93 for secondary infection).

**Table 4:** Sensitivity of NS1 assays in patients with primary and secondary serological profiles.

		SD NS1	Bio-Rad NS1	CTK NS1	SD NS1 or IgM	SD NS1 or IgM or IgG^b^	CTK NS1 or IgM	CTK NS1 or IgM or IgG	Sampling day^c^	Ct^d^
Status	Total (N)	% Sensitivity							Median (range)	
Primary infection	115	95.7% (110/115)	90.4% (104/115)	93.9% (108/115)	97.4% (112/115)	97.4% (112/115)	95.7% (110/115)	97.4% (112/115)	3 (0–7)	19.42 (12.6–37.73)
Secondary infection	10	60%^b^ (6/10)	50% (5/10)	60%^b^ (6/10)	90% (9/10)	100% (10/10)	90% (9/10)	100% (10/10)	4 (0–7)	29.93 (25.97–34.93)
*P* value^a^		0.002	0.003	0.005	0.287	1	0.4	1	0.584	0.02

^a^ Fisher's exact test was used to compare the sensitivity between primary and secondary infections.

^b^ Fisher's exact test was used to compare the sensitivity between SD/CTK NS1 and SD/CTK NS1 or IgM/IgG. Please refer to the text for details (60% *vs* 90%, P = 0.303; 60% *vs* 100%, P = 0.087).

^c^ Sampling day; days post-symptom onset.

^d^ Mean Ct values (range) of dengue qRT-PCR assay.

### Influence of IgM and IgG antibodies on the performance of NS1 rapid tests

The sensitivity of the NS1 RDT is influenced by the presence of DENV-reactive antibodies [15]. Thus, the presence of anti-DENV IgM and IgG antibodies was determined using capture IgM and IgG ELISAs [23], and the effect of dengue IgM and IgG antibodies on the performance of NS1 RDTs was analyzed in this study. The presence of IgM in acute-phase samples influenced the sensitivity of all of the NS1 RDTs without significant differences (Table 5). However, the presence of IgG in IgM-negative samples reduced the sensitivity of all of the NS1 rapid tests to a greater extent. Furthermore, the presence of both IgM and IgG in serum samples significantly reduced the sensitivity of all of the NS1 rapid tests.

**Table 5:** Influence of anti-DENV IgM or IgG on the sensitivity of dengue NS1 rapid tests.

	SD				Bio-Rad				CTK			
ELISA^a^	IgM-/IgG+	IgM+/IgG+	IgM-/IgG-	IgM+/IgG-	IgM-/IgG+	IgM+/IgG+	IgM-/IgG-	IgM+/IgG-	IgM-/IgG+	IgM+/IgG+	IgM-/IgG-	IgM+/IgG-
Sensitivity^b^	66.7% (4/6)	45.5% (5/11)	93.9% (93/99)	100% (19/19)	66.7% (4/6)	36.4% (4/11)	90.9% (90/99)	89.5% (17/19)	66.7% (4/6)	54.5% (6/11)	91.9% (91/99)	100% (19/19)
Dengue cases	6	11	99	19	6	11	99	19	6	11	99	19

^a^ Results of the capture IgM and IgG ELISAs.

^b^ The sensitivity is presented as a % (number of positive NS1 results/number of positive qRT-PCR results).

### Comparison of anti-DENV IgM and IgG RDTs using capture IgM and IgG ELISAs as references

To evaluate the specificity and sensitivity of the CTK and SD IgM/IgG RDTs, we used capture IgM and IgG ELISAs as a standard assay (Table 6). The specificity and sensitivity of the CTK RDT were lower than those of the SD IgM RDT, without a significant difference. The sensitivity of the SD IgG RDT was lower than that of the CTK RDT (*P* = 0.175), while the specificity of the CTK RDT was significantly lower than that of the SD RDT (*P*<0.001).

**Table 6:** Anti-DENV IgM/IgG RDT compared with capture IgM/IgG ELISA as a standard assay.

Method	IgM ELISA			IgG ELISA		
	Sensitivity	Specificity	Kappa value	Sensitivity	Specificity	Kappa value
SD RDT	76.7% (23/30)	68.6% (72/105)	0.3455 (0.1947–0.4963)	70.6% (12/17)	89.1% (106/119)	0.4965 (0.2981–0.6949)
CTK RDT	73.3% (22/30)	61% (64/105)	0.2462 (0.1028–0.3896)	94.1% (16/17)	50.4% (60/119)	0.1809 (0.0855–0.2763)
*P* value^a^	1	0.312		0.175	<0.001	

^a^ Fisher's exact test.

### Sensitivity of the NS1 RDT in the detection of the different serotypes of DENV

To understand whether the NS1 RDTs used in this study performed differently when detecting the different serotypes of DENV at the acute stage, the sensitivities of three RDTs were analyzed, and the results are listed in Table 7. The results showed that the three RDTs had the highest sensitivity for DENV-1 detection compared to the sensitivities for the other serotypes of DENV. The sensitivities were lowest for DENV-4 detection, especially when the Bio-Rad and SD NS1 RDTs were used for detection. The Bio-Rad NS1 RDT showed a similar sensitivity in detecting DENV-1 and DENV-3, while the RDTs were significantly less sensitive for DENV-2 (72.7%) and DENV-4 (33.3%) detection (P<0.05, Fisher’s exact test). The SD NS1 RDT had significantly higher sensitivity for the detection of DENV-1 than for DENV-2 or DENV-3. When the CTK NS1 RDT was used, the difference was not significant among the four serotypes, although the sensitivity was highest for DENV-1 detection followed in order by DENV-2/3 and DENV-4 detection.

**Table 7:** Sensitivity of the three NS1 RDTs in the detection of the different serotypes of DENV.

	DENV-1	DENV-2	DENV-3	DENV-4
SD	100% (44/44)	84.1% (37/44)	88.6% (39/44)	33.3% (1/3)
Bio-Rad	95.5% (42/44)	72.7% (32/44)	90.9% (40/44)	33.3% (1/3)
CTK	95.5% (42/44)	86.4% (38/44)	86.4% (38/44)	66.7% (2/3)
*P* value^a^	0.357	0.217	0.798	0.638

^a^ Chi-square test.

## Discussion

DENV NS1 antigen is secreted in the sera of patients during acute DENV infection. Despite its role in dengue genome replication, the possible pathogenic effects of NS1, as well as its antibodies, have been documented in severe dengue disease. DENV NS1 has been shown to increase the secretion of macrophage migration inhibitory factor (MIF), resulting in upregulation of glycocalyx degradation factor heparanase 1 (HPA-1) and degradation of endothelial glycocalyx, as well as endothelial autophagy, leading to endothelium hyperpermeability [26–28]. In addition, activation of the Toll-like receptor 4 [29] and p38 MAPK [30] pathways by NS1 and endocytosis of NS1 [31] have been noted in endothelium hyperpermeability. Anti-NS1 antibodies cross-react with human endothelial cells and cause NO-mediated apoptosis of these cells [32, 33] via ceramide-regulated glycogen synthase kinase-3 beta and NF-kappa B activation [34]. Moreover, anti-NS1 antibodies cause thrombocytopenia in severe dengue disease by enhancing platelet phagocytosis by macrophages [35].

We evaluated the specificity and sensitivity of commercial RDTs for DENV NS1 and IgM/IgG detection with a serum panel collected from donors with suspected dengue fever during the 2012–2013 dengue outbreak in Kaohsiung City, Taiwan. The results suggested that the sensitivities of the three commercial RDTs were not statistically different for the detection of DENV NS1 in acute-phase sera from qRT-PCR-positive patients. The SD NS1 RDT results showed the highest agreement with the reference qRT-PCR results, followed by the Bio-Rad NS1 RDT results when the specificity was considered according to the Kappa statistics. The inclusion of an anti-DENV IgM antibody in both the CTK and SD NS1 RDTs increased the sensitivity, while the specificity of the tests was reduced. The inclusion of the anti-DENV IgM and IgG antibodies increased the sensitivity of the CTK and the SD NS1 RDTs, while the specificity of the CTK RDT was remarkably reduced. The detection sensitivity was significantly increased when results of the capture IgM and IgG ELISA were combined with the Bio-Rad NS1 RDT results, compared with the detection sensitivity of the Bio-Rad NS1 RDT results alone or including the anti-DENV IgM ELISA results. Some of the 37 OFI samples were positive when tested with the NS1 RDTs. One interpretation is that these are false-positive results occurred because these samples were DENV RNA-negative. Conversely, it has been shown that the DENV NS1 antigen remains positive after the DENV RNA disappears due to the long half-life of the NS1 protein [12, 36]. Moreover, the possibility that the negative qRT-PCR results could have resulted from the presence of PCR inhibitors cannot be excluded.

We further analyzed whether the sampling time affected the sensitivity of the NS1 RDTs. The results suggested that the sensitivities of the Bio-Rad NS1 RDT were lower than those of the CTK and SD NS1 RDTs when used to test both samples obtained within three days post-symptom onset or those collected later, although the difference was not significant. When tested with samples collected within three days after the onset of symptoms, the sensitivity of the CTK and SD rapid tests was significantly higher than that for those collected within three days after symptom onset. Fifty to eighty percent of IgG-positive samples were collected from secondary infection patients. Among these NS1-negative samples, 20% to 25% were IgG-positive samples, and 70% to 81.8% were IgM/IgG-positive samples. The sensitivity for samples collected within 3 days of PSO was higher than the sensitivity for samples collected after 3 days of PSO, consistent with the results of other studies [13, 37–39]. All of the NS1 RDTs were significantly more sensitive to primary DENV infections than to secondary infections. Among the ten secondary infection samples, ninety to one hundred percent were IgG-positive samples. Among the four NS1-negative secondary infection samples, three were IgM-positive, and all were IgG-positive. In addition, the results showed that the sensitivity of the NS1 RDT was generally relatively low when used to test sera with a serological status of IgM/IgG positive, followed by the samples with IgG-positive results. Among the NS1-positive samples, 83.4% were IgM/IgG-negative. Our results were consistent with the notion that the presence of anti-DENV IgG, detected using either RDT or ELISA, in a sample can decrease the sensitivity of the NS1 RDT [6, 24, 37, 40]. We also found increased NS1 sensitivity in samples with a measurable DENV-reactive IgM level, similar to the results of Tontulawat *et al*. [41]. Considering these results together, including the results of IgM or IgG, would definitely increase the ability to diagnose the acute phase of either primary or secondary DENV infection when the NS1 level is declining [15, 42, 43].

The sensitivity and specificity of both the CTK and SD IgM RDTs were not statistically different using capture IgM ELISA as a standard assay, and the analysis of the Kappa coefficient suggested fair agreement between both IgM RDTs and the IgM ELISA. The sensitivity of the CTK IgG RDT was higher than that of the SD IgG RDT, but the difference was not significant because the sample size was small. However, the specificity of the SD IgG RDT was significantly higher than that of the CTK IgG RDT. The Kappa coefficient analysis suggested moderate agreement between the IgG ELISA and SD IgG RDT results, while the agreement between the IgG ELISA and CTK IgG RDT results was very poor.

Previous studies have reported that the sensitivity of the CTK and SD NS1 rapid tests ranged from 44% to 87% [15, 44, 45] and that the NS1 RDTs showed lower sensitivity for secondary infections than for primary infections [15, 24, 45–47]. The high sensitivity of the three NS1 RDTs investigated in this study might be because 92% of the sera were collected from patients with primary infections. These results suggested that the sensitivity was decreased when the NS1 RDT was applied to the convalescent phase samples or to samples collected from patients with secondary infections. However, for DENV-4, we had only 3 samples. The sensitivities of the three RDTs were 95.5%-100% (DENV-1), 72.7%-86.4% (DENV-2), 86.4%-90.9% (DENV-3) and 33.3%-66.7% (DENV-4) for each of the four serotypes. The sensitivity of the SD and CTK NS1 RDTs for DENV-3 detection was similar to that in our previous study, in which the sera were collected from a 2013 DENV-3 dengue outbreak in the Solomon Islands (S1 Table) [24]. However, the inclusion of anti-DENV IgG in the CTK NS1 RDT largely decreased the specificity, which might be due to the assay being very sensitive to the presence of low levels of IgG induced in previous DENV infections, or the assay might cross-reactive with the IgG induced by vaccination (e.g., JEV in Taiwan) [48, 49] or infection with other flaviviruses or malaria, especially in the Solomon Islands [50–52]. RT-PCR has been recommended for confirmational diagnosis of dengue virus infection, allowing for early intervention for surveillance, outbreak investigations, and clinical management. In our previous studies, reverse transcription insulated isothermal PCR (RT-iiPCR) using pan-DENV or singleplex DENV-1–4 serotyping reagents in a mobile semi-automated taco mini/Pockit system or a fully automated sample-to-answer POCKIT Central system were validated for rapid detection of dengue virus in human serum in approximately 90 minutes, reaching analytical sensitivity of 1 to 10 PFU/ml of serum for the four serotypes of dengue virus [53–55]. However, the disadvantages of the nucleic acid test are that serum samples must be shipped to a central laboratory, and the experiments must be performed by well-trained medical technician to detect DENV. These results together suggested that the DENV NS1 RDT used in this study was valuable for the rapid screening of acute DENV infection in 15 mins, at least for DENV-1, DENV-2 and DENV-3. From previous reports, the sensitivity of the NS1-based assay using RT-PCR as a reference method was relatively low for the detection of DENV-4, compared to the sensitivity for the detection of the other three serotypes [14, 20, 21]. Our study had similar findings, but since our study had a limited number of cases (3 samples), it was difficult to reach the conclusions of other studies. NS1 RDTs had great value for screening dengue cases in the face of a large dengue outbreak since they were applied routinely since the biggest dengue outbreak in Taiwan in 2015. However, the sensitivity of RDTs for DENV-4 from previous studies seemed lower than that for the other serotypes, ranging from 36% to 71% [14, 20, 21]. From February to June 2019 in Taiwan, the sensitivity of NS1 RDT (SD) for DENV-4 was 70% (14/20) from autochthonous RT-PCR positive dengue cases (unpublished data). For precise detection of DENV-4-infected dengue cases, concomitant RT-PCR usage should be important to increase the detection rate once a case is highly suspected.

## Conclusions

NS1 is a viral antigen associated with viremia in the acute phase of DENV infections. Although the detection of DENV by RT-PCR is widely accepted as the reference method for the diagnosis of DENV infection, NS1 antigens persist longer than DENV in the blood of infected patients, and the high sensitivity and specificity of the NS1 RDTs suggest that a user-friendly RDT could be an alternative and convenient tool. Factors including sample collection day, primary or secondary infection, and different DENV serotypes affect the performance of diagnostic RDTs. Our results suggest that the NS1 RDT is a good alternative tool for qRT-PCR for timely dengue disease management and prevention in dengue endemic regions where medical resources are lacking or during large dengue outbreaks.

## Supporting information

S1 Table Sensitivity and specificity of the current and our previous study. (DOCX)
