# Supplementary material for: Evaluation of rapid diagnostic tests to detect dengue virus infections in Taiwan
Source: PLoS One. 2020 Sep 29;15(9):e0239710. doi: 10.1371/journal.pone.0239710 (PMC7524001; doi:10.1371/journal.pone.0239710)
Supplement: S1 Table — (DOCX) [file pone.0239710.s001.docx]

**S1 Table. Sensitivity and specificity of the current and our previous study.**

|  | Sensitivity | | | Specificity | | |
| --- | --- | --- | --- | --- | --- | --- |
|  | Current study | | Liu *et al.* 2018[24] | Current study | | Liu *et al.* 2018[24] |
| Assay parameter | All serotypes | DENV-3 | DENV-3 | All serotypes | DENV-3 | DENV-3 |
| SD NS1 | 89.7% (122/136) | 88.6% (39/44) | 90.9% (220/242) | 91.9% (34/37) | - | 100% (170/170) |
| SD NS1+IgM | 95.6% (130/136) | - | 93% (225/242) | 89.2% (33/37) | - | 100% (170/170) |
| SD+IgM+IgG | 97.1% (132/136) | - | 93% (225/242) | 86.5% (32/37) | - | 100% (170/170) |
| CTK | 89% (121/136) | 86.4% (38/44) | 92.6% (224/242) | 73% (27/37) | - | 78.8% (134/170) |
| CTK+IgM | 95.6% (130/136) | - | 94.6% (229/242) | 70.3% (26/37) | - | 62.4% (106/170) |
| CTK+IgM+IgG | 97.8% (133/136) | - | 98.3% (238/242) | 43.2% (16/37) | - | 24.1% (41/170) |
